# Noncontrast magnetic resonance imaging versus ultrasonography for hepatocellular carcinoma surveillance (MIRACLE-HCC): study protocol for a prospective randomized trial

**DOI:** 10.1186/s12885-018-4827-2

**Published:** 2018-09-24

**Authors:** Chansik An, Do Young Kim, Jin-Young Choi, Kwang Hyub Han, Yun Ho Roh, Myeong-Jin Kim

**Affiliations:** 1 Department of Radiology, Research Institute of Radiological Science, Yonsei University College of Medicine, 50 Yonsei-ro, Seodaemun-gu, Seoul, 03722 South Korea; 2 Department of Internal Medicine, Yonsei University College of Medicine, Seoul, South Korea; 3 Biostatistics Collaboration Unit, Medical Research Center, Yonsei University College of Medicine, Seoul, South Korea

**Keywords:** Hepatocellular carcinoma, Surveillance, Magnetic resonance imaging, Ultrasonography

## Abstract

**Background:**

Biannual ultrasound (US)—with or without alpha-fetoprotein (AFP)—is recommended by current guidelines for the surveillance of hepatocellular carcinoma (HCC). However, the inadequate sensitivity of US has been a concern. Magnetic resonance imaging (MRI) is known to have high sensitivity in detecting hepatic malignancies, even without contrast enhancement. The purpose of our study is to compare US with noncontrast (unenhanced) MRI for HCC surveillance of high-risk patients.

**Methods/design:**

MIRACLE-HCC (usefulness of noncontrast MagnetIc Resonance imAging versus nonContrast ultrasonography for surveiLlancE of HepatoCellular Carcinoma) is a prospective, single-center, nonblinded, balanced-randomized, parallel-group study. This study was approved by our institutional review board, and informed consent will be obtained from all participating patients. All patients with compensated liver cirrhosis will undergo noncontrast US or MRI, with serum AFP testing every 6 months. If a suspicious lesion is newly detected, or if the serum AFP level is elevated in an increasing trend for two consecutive tests, dynamic contrast-enhanced imaging will be performed to confirm the diagnosis. The primary endpoints are detection rates of very early or early stage HCC, stage distribution at the initial diagnosis, and false positive referral rates, which will be compared using Fisher’s exact or chi-square tests. The study will include 416 patients in a tertiary academic medical center in South Korea.

**Discussion:**

MIRACLE-HCC is the first prospective randomized trial to compare the effectiveness of noncontrast MRI and noncontrast US in the surveillance of HCC in at-risk patients. The results of this trial will show whether noncontrast MRI surveillance is superior to noncontrast US surveillance in the early detection of HCC. The trial will also determine whether there are fewer false referrals with noncontrast MRI than with noncontrast US and, eventually, whether there is improvement in the overall survival of HCC patients.

**Trial registration:**

The date of trial registration (ClincalTrials.gov: NCT02514434) for this study is July 23, 2015. Enrollment of participants was finished in November 2017. No authors have relationships, conditions, or circumstances that present potential conflicts of interest.

**Electronic supplementary material:**

The online version of this article (10.1186/s12885-018-4827-2) contains supplementary material, which is available to authorized users.

## Background

Periodic surveillance of patients at high risk of developing hepatocellular carcinoma (HCC) is known to improve early tumor detection and overall survival [1–4]. Biannual noncontrast ultrasound (US)—with or without alpha-fetoprotein (AFP)—is recommended as a surveillance imaging modality by the current guidelines, mainly because of wide accessibility and low cost [5–8]. However, the inadequate sensitivity of US has been a concern [9], with a meta-analysis reporting a pooled sensitivity of 63% for detecting early stage HCC [10]. Moreover, the reported sensitivities of US vary widely—from 32 to 92%—in clinical practice [11, 12], demonstrating that US results can be greatly affected by several factors, including imaging protocols, equipment, patient characteristics, and operator experience [13–15].

Given the limited effectiveness of noncontrast US, other imaging modalities, such as contrast-enhanced US, contrast-enhanced computed tomography (CT), and contrast-enhanced magnetic resonance imaging (MRI), have been suggested for use in HCC surveillance [16–21]. However, contrast-enhanced US still has the limitation of being greatly influenced by patient characteristics (i.e., poor echogenic window or liver macro-nodularity) and operator experience, and CT may not be a desirable surveillance modality due to the potential risk of radiation exposure [22]. Although contrast-enhanced MRI is superior to noncontrast (unenhanced) US in detecting small tumors in patients with chronic liver disease [23], its use in HCC surveillance may be hampered by limited accessibility and high cost, as well as the issue of gadolinium-based contrast agent accumulation in human organs [24].

Even without contrast enhancement, MRI is likely to show higher sensitivity than US in detecting hepatic malignancies [25]. Furthermore, noncontrast MRI does not include dynamic sequences using contrast agents, thereby reducing the cost and scanning time, and avoiding the accumulation of gadolinium-based contrast media. We believe that these advantages could make noncontrast MRI a potential alternative to US for HCC surveillance. Therefore, we set up a prospective randomized trial to compare noncontrast US with noncontrast MRI in HCC surveillance of high-risk patients.

## Methods/design

### Study design and setting

The current trial (usefulness of noncontrast MagnetIc Resonance imAging versus nonContrast ultrasonography for surveiLlancE of HepatoCellular Carcinoma [MIRACLE-HCC]) is a prospective, single-center, nonblinded, balanced-randomized, parallel-group study. This trial is registered with ClinicalTrials.gov (identifier: NCT02514434), and the date of registration is July 23, 2015.

This study has been approved by our institutional review board (IRB) and will be conducted in accordance with the ethical principles stated in the Declaration of Helsinki and Good Clinical Practice guidelines. Any modifications to the protocol will require a formal amendment to the protocol and will be reviewed by the IRB. These modifications will be implemented only after they are approved by the IRB and notified to all participating investigators and patients. From August 2015, we recruited patients who started or had been under surveillance for HCC at the liver cancer center of Severance Hospital, a 2260-bed academic tertiary referral hospital in Seoul, South Korea. Before inclusion in the study, informed written consent has been obtained from each patient. The last patient was enrolled on November 29, 2017, and follow-up to see primary and secondary endpoints is still ongoing.

In South Korea, whose total population is approximately 51 million, the crude incidence rate of liver cancer in 2013 was 32 per 100,000 [26], and 62.7% of HCC cases are attributable to chronic hepatitis B virus infection [27]. In Korea, liver cancer is the second-most common cause of death from cancer and was estimated to be responsible for approximately 11,560 deaths in 2014 and ranked first in terms of the total economic burden during 2001–2010 [28, 29].

### Eligibility criteria

Eligible participants are adults aged 20–70 without history of liver cancer who are at risk of developing primary hepatic carcinoma (hepatitis B surface antigen positive [HBsAg+] or anti-hepatitis C virus positive [anti-HCV+] with cirrhosis, or cirrhosis from any causes), who have preserved liver function (Child-Pugh class A), and for whom the absence of a liver tumor has been confirmed clinically (no symptoms and no elevated tumor marker) and radiologically (on US, CT, or MRI) at the time of screening. Exclusion criteria are diagnosis of malignancy in the last 5 years, possible pregnancy, and severe cardiovascular, respiratory, renal, or infectious disease. The presence of cirrhosis will be determined based on the histologic or radiologic findings, including liver surface nodularity, margin blunting, segmental hypertrophy/atrophy, and findings secondary to portal hypertension (i.e., splenomegaly, esophageal or gastric varices, ascites, or reverse portal vein flow).

### Participant flow

**Fig. 1:**
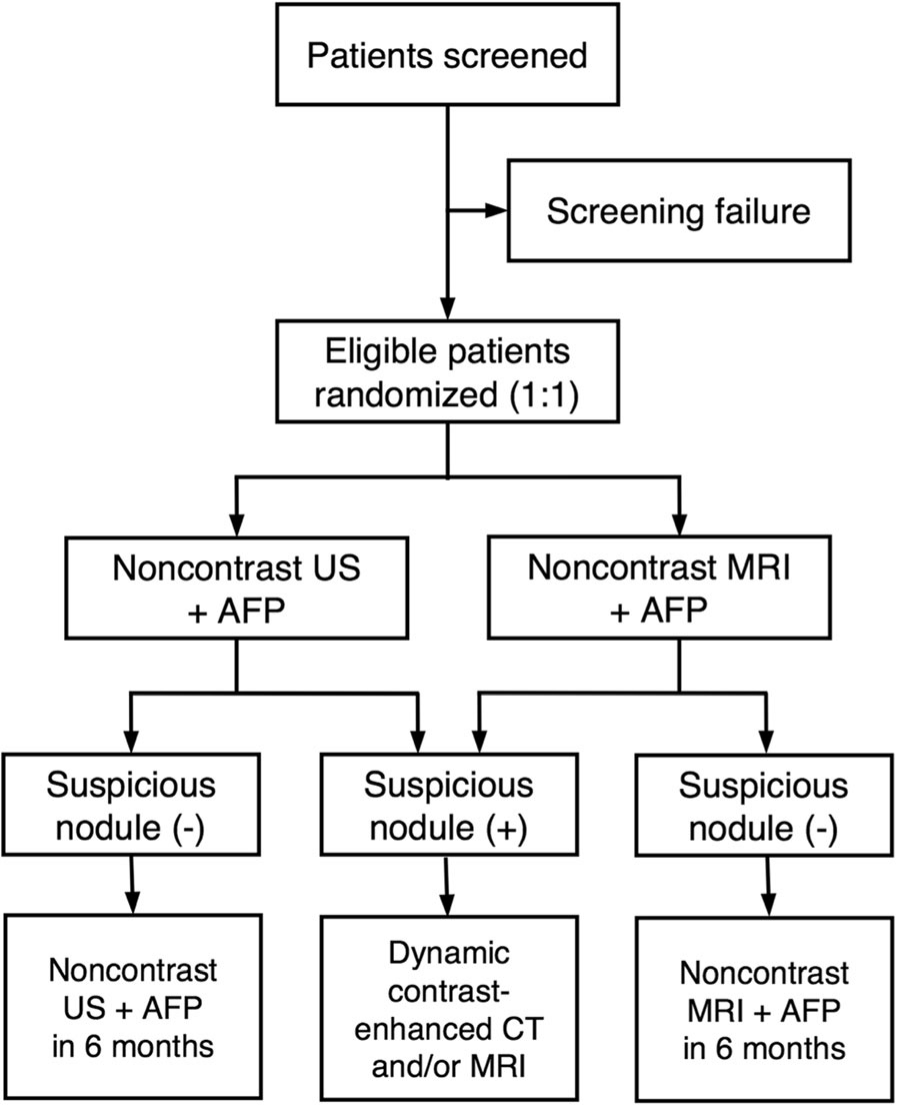
Participant flow

A trained research nurse will introduce the trial to potentially eligible patients, who will be provided with detailed information about the trial. They will then be able to have an informed discussion with participating physicians, who will obtain written consent if the patients agree to participate in the trial. Enrolled participants will be randomly assigned to either abdominal US or noncontrast MRI in addition to serum AFP testing for HCC surveillance (Fig. 1). They will be evaluated by at least 10 rounds of surveillance tests at 6-month intervals (with a variation of ±1 month allowed). The first surveillance test will be performed 6 months after the most recent imaging study showing no evidence of hepatic malignancy. If a new lesion detected during US or MRI surveillance is suspected to be malignant according to the predefined criteria (see Imaging evaluation section) or if the serum AFP level is elevated in an increasing trend for two consecutive tests, dynamic contrast-enhanced CT will be performed [6, 30]. The reference value for AFP is less than 9 ng/mL at our institution. Additional dynamic liver MRI may be performed at the discretion of clinicians. If the surveillance test result is determined to be a false positive by subsequent tests, the patient will return for the next scheduled surveillance test. If a patient is confirmed as having a hepatic malignancy, the patient will receive standard treatment according to the guidelines [5–8]. We will continue follow-up of treated patients for at least 5 years to obtain and compare the overall survival rates.

Unscheduled examinations may be performed under various circumstances. For example, a participant may visit the hospital and undergo dynamic contrast-enhanced CT between surveillance tests because of symptoms such as abdominal pain or hematemesis. We will record and report all such cases of unscheduled visits and examinations.

A patient will be considered a dropout, if that participant 1) withdraws consent, 2) receives liver transplantation without diagnosis of liver cancer, 3) is diagnosed as having malignancies other than liver cancer, or 4) does not take two or more consecutive surveillance tests during the trial. To minimize dropout, we will periodically contact participants to remind them of their next scheduled surveillance and to encourage participation. We will also make efforts to obtain relevant information from patients lost to follow-up; for example, if a patient is transferred to a different hospital after hepatic malignancy is diagnosed through this trial, we will contact the patient and ask for information on the results of follow-up treatment or pathological diagnosis.

### Ultrasonography

Abdominal noncontrast US will be performed using commercially available machines (Pro-Sound Alpha10 or Pro-Sound F75, Hitachi Aloka Medical, Tokyo, Japan; ACUSON S2000, Siemens Medical Solutions, Mountain View, CA, USA; iU22, Philips Medical Systems, Best, The Netherlands) with 5-MHz curved-array transducers. Instead of designating specific investigators to perform surveillance US for this trial, we will perform surveillance US as in our current clinical practice. At our institution, surveillance US is performed by well-trained hepatology fellows. They are trained before they start practicing abdominal US, and during the first month of practice, they perform US under the guidance and supervision of faculty members. In performing US, operators follow the guidelines of the Korean Society of Ultrasound in Medicine [31].

### MRI

MRI will be performed using four 3.0-T systems (MAGNETOM Trio Tim, Siemens Healthcare, Erlangen, Germany; Prisma Fit, Siemens Healthcare; Discovery MR 750, GE Healthcare, Milwaukee, WI, USA; Ingenia CX, Philips Healthcare, Best, the Netherlands). All images will be obtained in the transverse plane with a field of view of 44 cm × 33 cm or 40 cm × 30 cm, according to the patient’s body size. After obtaining the localizer images (scanning time: ~ 20 s), T1-weighted images will be obtained by dual-echo T1-weighted gradient-recalled echo (GRE) (~ 20 s) and three-dimensional volume interpolated GRE (~ 20 s) sequences. T2-weighted images will be acquired by single-shot fat-suppressed navigator-triggered fast or turbo spin echo sequence (3–4 min, depending on the regularity of respiration and patient compliance) and by single-shot breathhold fast or turbo spin echo sequence with a long echo time of 140–150 msec (scanning time: ~ 20 s). We will also obtain free breathing or respiratory gated diffusion-weighted images with b-values of 50, 400, and 800 s/mm^2^ (2–4 min), respectively. The apparent diffusion coefficient will be automatically calculated and displayed as a corresponding map. The total scanning time will be approximately 7–10 min, depending on the MRI machine, and the regularity of patients’ respiration and their compliance. Details regarding MRI parameters are presented in Table 1.

**Table 1:** Parameters of surveillance noncontrast MRI

Sequence	Scanner^a^	Matrix Size	ST (mm)	Gap (mm)	TR (msec)	TE (msec)	FA (°)
Dual-echo T1-WI GRE	Magnetom (2D)	256 × 192	6	1.2	150	1.23/2.46	65
	Ingenia (3D)	278 × 256	2	0	3.2	1.15/2.30	10
	Discovery (3D)	320 × 256	2	0	3.9	1.12/2.35	12
	Prisma (3D)	320 × 256	2	0	9	1.34/2.73	9
T1-WI 3D GRE	Magnetom	256 × 192	2	0	2.54	0.95	13
	Ingenia	256 × 282	2	0	3.1	1.42	10
	Discovery	320 × 288	2	0	4.2	1.9	12
	Prisma	320 × 256	2	0	2.68	1.08	9
T2-WI with fat saturation	Magnetom	256 × 192	4	1	466	96	150
	Ingenia	320 × 212	4	1	758	80	90
	Discovery	320 × 256	4	1	2800	80	90
	Prisma	320 × 182	4	1	620	105	107
T2-WI with long TE	Magnetom	320 × 168	4	1	450	148	150
	Ingenia	320 × 186	4	1	522	150	90
	Discovery	320 × 224	4	1	840	150	90
	Prisma	320 × 208	4	1	600	153	98
DWI	Magnetom	128 × 96	6	1	5200	67	90
	Ingenia	128 × 128	5	1	4848	55	90
	Discovery	128 × 80	5	1	4800	51	90
	Prisma	140 × 112	5	1	5500	63	90

^a^Full scanner names are Magnetom Trio Tim (Siemens), Ingenia CX (Philips), and Discovery MR750 (GE), and Prisma Fit (Siemens)

*2D* Two-dimensional, *3D* Three-dimensional, *ST* Slice thickness, *TR* Repetition time, *TE* Echo time, *FA* Flip angle, *GRE* Gradient-recalled echo, *DWI* Diffusion-weighted imaging

### Imaging evaluation

Surveillance US findings will be interpreted and reported by the well-trained hepatology fellows. During US, a suspicious nodule on US is defined as a newly appearing nodule > 1 cm. Diffuse infiltrative lesions with or without suspected tumor in the vein are also considered suspicious for HCC. Simple cysts or lesions that have been previously diagnosed as benign lesions, such as hemangiomas, and show no significant interval change are not considered suspicious, irrespective of the size.

Surveillance noncontrast MRI will be evaluated by one of three board-certified abdominal radiologists (MJK, JYC, and CA). At noncontrast MRI, a suspicious nodule is defined as a newly appearing nodule > 1 cm that shows at least one of the following imaging features: T1 hypointensity, T2 hyperintensity, diffusion restriction, nodule-in-nodule pattern (mosaic appearance), iron sparing, heterogeneous fatty changes, blood products, or definite tumor diameter increase. Diffuse infiltrative lesions with or without a suspected tumor in the vein are also considered suspicious. Simple cysts, hemangiomas, and lesions that have been previously diagnosed as benign lesions are not considered suspicious, irrespective of the size.

Diagnostic dynamic contrast-enhanced CT or MRI will be evaluated and reported using the Liver Imaging Reporting and Data System (LI-RADS) [30], by one of the three board-certified abdominal radiologists (MJK, JYC, and CA). LI-RADS version 2014 was used at our institution until June 2017; after that date, we have been using LI-RADS version 2017.

### Reference standards

The final diagnosis will preferably be determined based on histopathologic findings through surgery (when appropriate, according to the practice guidelines) or biopsy. When histopathologic diagnosis is not possible (i.e., if a patient refuses or cannot tolerate an invasive procedure) or when invasive biopsy is deemed to do more harm than good (i.e., a patient with radiologically definite HCC at an advanced stage in whom curative treatment is not indicated), radiologic diagnosis will be applied. For radiologic diagnosis, lesions categorized as LR-5, LR-5 V (LR-TIV in LI-RADS version 2017), or LR-M according to the LI-RADS diagnostic algorithm will be diagnosed as hepatic malignancies [30].

### Outcomes

The primary endpoints are detection rates of very early or early stage HCC, stage distribution at the initial diagnosis, and false positive referral rates. The detection rate is defined as the number of patients whose liver cancer is detected using a given surveillance modality divided by the total number of patients under surveillance with the modality, expressed as a percentage. Very early stage cancer is defined as a single tumor < 2 cm, and early stage cancer is defined as a single tumor < 5 cm or up to three tumors < 3 cm [7, 8]. Surveillance is considered a failure when patients are diagnosed at late (i.e., intermediate or advanced) stages. The false positive rate is defined as the number of positive tests that are eventually confirmed as negative on subsequent dynamic imaging and/or pathologic examination divided by the total number of tests in a specific surveillance modality, expressed as a percentage. The secondary endpoints are 5- and 10-year overall survival rates. We will report these long-term outcomes in separate papers.

### Sample size

Based on the past prevalence of HCC in at-risk patients undergoing surveillance in our institution, we anticipate that the prevalence of HCC in our study cohort will be approximately 18% over a study period of 5 years. The diagnostic sensitivities of noncontrast US and noncontrast MRI are expected to be 60% and 90%, respectively [10, 25]. Based on these expected prevalence and sensitivities, and an anticipated dropout rate of 15%, we calculated that we would need a sample size of 416 patients (208 for each group) to yield 80% power (1 minus the probability of a type II error) to detect a significant difference in detection rate between the noncontrast US and noncontrast MRI arms, with a two-sided type I error of 5% [32].

### Randomization and blinding

A research nurse will assign participants into either the noncontrast US group or the noncontrast MRI surveillance group according to a computer-generated randomization list. The randomization sequence will be created using SAS 9.2 (SAS Institute, Inc., Cary, NC, USA) statistical software by an investigator with no clinical involvement in the trial, with a 1:1 allocation using a random block size of 4. Only the research nurse will have knowledge of the randomization sequence. However, once investigators obtain a participant’s consent and the research nurse assigns him or her to the noncontrast US or noncontrast MRI group, the participant and investigators will inevitably be aware of the surveillance assignment at the time of the initial surveillance examination. Therefore, blinding of participants and investigators is not possible for this trial.

### Data management

We will use the Electronic Case Reporting System with Accuracy, Safety, and Efficacy (e-CASE)—a web-based case report form (https://ecase.yuhs.ac)—for data management. The research nurse will enter data into the e-CASE, and a trained data manager will periodically check the accuracy and completeness of the entered data. The data will be kept in the archive of the data center, which has built-in security features preventing unauthorized access. Only investigators, a research nurse, and an independent monitor who will perform data monitoring and auditing will have access to the full dataset. Personal information about participants will be coded and depersonalized.

### Statistical analysis

Both intention-to-treat (considering all patients as randomized irrespective of the surveillance test they actually receive) and per-protocol analyses will be performed. The detection rate, stage distribution, and false positive rates will be compared between the noncontrast US and noncontrast MRI arms using the Fisher’s exact or chi-square test. To compare the variables between the two groups, we will use the independent t-test or Mann-Whitney U test for continuous variables and the chi-square or Fisher exact test for categorical variables. If necessary, we will perform logistic regression analysis to determine the independent associations between variables. Survival will be compared using the Kaplan-Meier methods with log-rank test. Cox proportional hazard regression will be performed to determine the independent associations between survival and potential explanatory variables.

Subgroup analyses will be performed. We will perform the analyses after excluding patients in whom liver cancer is diagnosed at their first MRI surveillance tests following the negative US prior to study enrollment. We will compare the tumor stages between patients diagnosed at the first tests and those diagnosed at later tests among patients undergoing noncontrast MRI surveillance. These analyses are intended to examine and exclude the possibility that the tumors already exist but are missed by the noncontrast US test prior to enrollment. In addition, we will examine the effects of the quality of the echogenic window to the liver and the parenchymal nodularity on our primary endpoints. Furthermore, we will explore the additional benefit of serum AFP testing in conjunction with noncontrast US or noncontrast MRI for the detection of early stage HCC. Two-sided *p*-values < 0.05 are considered statistically significant. All statistical analyses will be performed using the SAS 9.2 software package.

### Monitoring

Interventions used for this trial are US and MRI, which are the widely used imaging tests whose adverse events are reported very rarely [33]. Furthermore, in this trial, these imaging tests will be performed without the use of contrast media. Therefore, there is no harm particularly anticipated from this study other than the inherent minimal risks of the imaging tests themselves. Nevertheless, we will monitor and record any adverse events occurring after study enrolment at every visit. Any serious adverse event will be reported to our IRB. As this study pose a minimal risk to participants as stated above, we will nominate a physician independent of this study as an independent safety monitor (ISM), but there will not be a formal data and safety monitoring committee. The ISM will review annually the trial processes and accumulating data, and determine if the trial should be modified or discontinued.

## Discussion

Our goal is to determine whether surveillance with noncontrast MRI can further improve the overall survival of patients at high risk of developing HCC, compared with surveillance using noncontrast US. To achieve this goal, we have designed this study as a two-arm randomized trial. However, no previous study has investigated the performance of noncontrast MRI in detecting HCC patients at early stages in a surveillance setting, although one study examined the diagnostic performance of noncontrast MRI to detect and diagnose HCC in a limited cohort of patients, primarily at very early or early stages [25]. Thus, we first set our primary endpoints as detection rate of early-stage HCC and false positive rate. At long-term follow-up of the current study cohort, we may observe a significant difference in overall survival between the two arms; if this is not possible, an appropriate sample size could be calculated based on the results of current trial for a future large-scale, multicenter trial.

We will perform surveillance every 6 months using both serum AFP and imaging tests, since the combined use of noncontrast US and AFP is recommended for surveillance in many practice guidelines published in Asia, including Korea [5, 6]. Inclusion of AFP may be a confounding factor in comparing the two imaging modalities. However, it would be unethical to exclude serum AFP testing from HCC surveillance for the sake of a clinical trial because the current evidence points to noncontrast US combined with serum AFP as the most effective surveillance strategy [34]. One advantage of this study design, however, is that this trial will allow us to assess the benefit of AFP performed in addition to US or MRI in HCC surveillance, by examining the proportion of cases where early-stage HCC is detected by AFP elevation with negative US or MRI results.

We will not designate experienced radiologists specifically for our surveillance US. Instead, as is currently practiced at our institution, well-trained hepatology fellows will perform surveillance US. In real-world clinical settings, many patients receive their surveillance US examinations in local community clinics instead of tertiary care centers, often by less-experienced US operators with less-than-optimal protocols or equipment [35]. It would be unrealistic to have all surveillance US examinations performed exclusively by a limited number of highly experienced operators.

MIRACLE-HCC is the first clinical trial to compare the effectiveness of noncontrast MRI and noncontrast US in the surveillance of HCC in at-risk patients. The results of this trial will show whether noncontrast MRI surveillance is superior to noncontrast US surveillance in the early detection of HCC, whether it results in fewer false referrals, and whether it can eventually improve the overall survival of HCC patients.

## Additional file

Additional file 1: Explanation for study participants (Translated version). This document describes the purposes, procedures, benefits, risks, inconveniences, and precautions of this study. All the patients read this document and fully understood the explanation of the study, before they decided whether or not they would agree to participate in the study. This document is a translated version from the original one written in Korean. (DOCX 22 kb)

## Abbreviations

AFP: Alpha-fetoprotein
CT: Computed tomography
e-CASE: Electronic Case Reporting System with Accuracy, Safety, and Efficacy
GRE: Gradient-recalled echo
HBsAg: Hepatitis B surface antigen
HBV: Hepatitis B virus
HCC: Hepatocellular carcinoma
HCV: Hepatitis C virus
IRB: Institutional review board
ISM: Independent safety monitor
LI-RADS: Liver Imaging Reporting and Data System
MIRACLE-HCC: Noncontrast magnetic resonance imaging versus noncontrast ultrasonography for surveillance of hepatocellular carcinoma
MRI: Magnetic resonance imaging
NEX: Number of excitations
US: Ultrasonography
