# Supplementary material for: Noncontrast magnetic resonance imaging versus ultrasonography for hepatocellular carcinoma surveillance (MIRACLE-HCC): study protocol for a prospective randomized trial
Source: BMC Cancer. 2018 Sep 24;18:915. doi: 10.1186/s12885-018-4827-2 (PMC6154402; doi:10.1186/s12885-018-4827-2)
Supplement: Supplementary file 1 — Explanation for study participants (Translated version). This document describes the purposes, procedures, benefits, risks, inconveniences, and precautions of this study. All the patients read this document and fully understood the explanation of the study, before they decided whether or not they would agree to participate in the study. This document is a translated version from the original one written in Korean. (DOCX 22 kb) [file 12885_2018_4827_MOESM1_ESM.docx]

**Explanation for study participants (Translated version)**

Study title: Usefulness of non-contrast magnetic resonance imaging versus non-contrast ultrasonography for surveillance of hepatocellular carcinoma: prospective randomized study

Study director: Professor Do Young Kim, Department of Internal Medicine, Yonsei University Health System, Severance Hospital, Yonsei-Ro 50-1, Seodaemun-Gu, Seoul City (Zip code:120-752) Phone: 02-2228-1992

This document describes the purposes, procedures, benefits, risks, inconveniences, and precautions of this study. You must read and fully understand the explanation of the study, before you decide whether or not you agree to participate in the study.

**Background and purpose of the study:**

Hepatocellular carcinoma (HCC) is one of the most serious consequences and the leading causes of death in patients with chronic liver disease. It is very important to perform periodic surveillance tests for early diagnosis of HCC, because the prognosis of the patient is different significantly depending on whether or not HCC is diagnosed at early stages in which curative surgery or liver transplantation is possible. According to the current Korean and other guidelines, abdominal ultrasonography every 6 months is recommended for patients at high risk of developing of HCC. Ultrasonography is a non-invasive, simple, patient-friendly and relatively inexpensive test, so it is used as a standard test for HCC surveillance. However, there is a limitation that ultrasonography is highly influenced by the operators’ experiences, and it shows disappointing abilities in detecting small HCCs especially in patients with severe nodular liver cirrhosis or atrophied liver, obese patients, or patients with diffuse type HCC. According to a recently published study, the sensitivity of abdominal ultrasonography in detecting early stage HCC (which is the target of surgery or liver transplantation) is as low as 60%. This result suggests that we need a more accurate imaging test than ultrasound for detecting HCCs at earlier stages. In a recently published study, computed tomography (CT) and magnetic resonance imaging (MRI) have been reported to be more sensitive in detecting early stage liver cancer than ultrasonography. However, CT is not suitable as a surveillance test due to problems such as radiation exposure and potential deterioration of kidney function caused by the use of contrast agent. MRI does not pose a risk of radiation exposure, but its high cost may be a limiting factor. However, the use of non-contrast MRI (hereinafter referred to as non-contrast MRI) may be a potential alternative to ultrasonography for the early detection of HCC because the cost can be similar to that of ultrasonography. The ability of non-contrast MRI has not yet been validated; thus, the purpose of this study is to examine the ability of non-contrast MRI as a surveillance imaging test for the early detection of HCC.

**Number of study participants and study period:**

This study will involve a total of 416 patients at high risk of developing HCC, except for those with previous history of other cancers in recent 5 years and pregnant or lactating women. The study period is a total of 5 years. If you agree to participate in the study, you will undergo surveillance tests every 6 months for the next 5 years.

**Description of process and procedure:**

If you decide to participate in the study, you will be asked to sign the consent form. After signing the consent form, you will receive a pre-scheduled ultrasonography to confirm that you have no liver cancer, and then you will go through basic cardiologic, hematologic, and thoracic examinations. Once you decide to participate in the study, you will be randomly assigned to one of two groups: ultrasonography surveillance test group and MRI surveillance test group. After that, you will undergo a surveillance test every 6 months with ultrasound or MRI, together with a tumor marker test, for the next 5 years. If a surveillance test reveals suspected liver cancer, you will receive a dynamic contrast-enhanced CT scan within a month for definite diagnosis. If it confirms the diagnosis, you will receive the standard treatment according to the guidelines. In other words, dynamic contrast-enhanced CT (Liver dynamic CT) will be taken when the liver mass is suspected in ultrasonography or non-contrast MRI. If a dynamic contrast-enhanced CT scan reveals no cancer, the surveillance test will be performed with the same imaging test every 6 months. If liver cancer is confirmed by dynamic CT scan, you will receive the standard treatment according to the guidelines. In this study, additional samples such as blood are not collected for the purpose of research other than this study.

**Costs related to study participation:**

Since this is a study for patients at high risk for liver cancer that are supposed to be screened every 6 months, you will be responsible for the costs of surveillance tests. Because non-contrast MRI scans do not use contrast agents, there is no extra cost for contrast agents. In our institution, the cost of non-contrast MRI is similar to that of abdominal ultrasonography.

**Benefits from participating in this study:**

According to the recently published studies, the sensitivity and specificity of ultrasonography exceed 90% when analyzed for liver cancer of all stages. However, the sensitivity to detect early liver cancer, which is the target of curative treatment such as surgery or transplantation, was reported to marginally exceed 60%. On the other hand, the sensitivity of non-contrast MRI in the diagnosis of liver cancer was 91.7% and the specificity was 77.5% in a study with patients undergoing curative surgery. Therefore, this study tries to verify the hypothesis that non-contrast MRI is more objective than ultrasonography which can be influenced by the operator's experience and patient's condition, and more sensitive and specific in the early detection and diagnosis of liver cancer, and ultimately more cost-effective than ultrasonography. Still, even if you are assigned to the ultrasound test group, it means that you will receive the standard care recommended by the current guidelines. If you are assigned to the non-contrast MRI group, liver cancer may be detected earlier if our hypothesis is correct.

**Risks, adverse effects, and discomfort in participating in this study:**

There is no large-scale prospective study to date on the role of non-contrast MRI as a surveillance test. However, considering the results of previous small-scale retrospective studies, there is little chance that non-contrast MRI is inferior to ultrasound in the early detection of liver cancer. Rather, if you undergo a surveillance test with MRI, it is highly likely that liver cancer is diagnosed at early stages where the cancer can be treated and cured. MRI could do harm if there is metallic devices in your body or if you has claustrophobia; however, we always check these possibilities before MRI. Therefore, there is no serious risk anticipated from participating in this study, and thus we do not have a specific plan for compensation.

**What the study participant should follow:**

If you are participating in this study, you should undergo surveillance tests at an interval of six months in accordance with the standard practice guidelines for management of HCC.

**Confidentiality:**

Your records, collected during your participation in this study, will be kept confidential during and after the study. Even when the results of this study are reported, presented, or published, your identity will be kept confidential. In order to verify the reliability of the procedures and data of the study within the scope of the relevant regulations without violating your privacy, the persons who monitors/examines this study, the heads of Institutional Review Board and the Korean Food and Drug Administration, are allowed to directly view your medical records. By signing this form, you or your legal representative will agree to allow them to view these records directly under such circumstances. In addition, your personal information collected in this study will not be provided to others nor will not be used secondarily.

**Voluntary participation and early interruption of study:**

You are voluntarily involved in this study. Therefore, if you withdraw your consent, your participation in the study will be discontinued. You may withdraw your consent to participate in this study at any time. In addition, we will notify you or your representative in a timely manner, when there is new information that may affect your ongoing research participation. Even if you decide not to participate in this study, you will continue to receive the best care as before, without affecting the quality of your future care. If you discontinue to participate in this study early, all data, including personal information collected in this study, will be discarded. However, if you withdraw your consent to participate in the study but still consent to the use and analysis of the data already collected, the collected data will not be discarded.

**Contact information:**

Please contact the following researchers if you have any questions about this study or if you think you have any research-related injury.

Researcher Name: Do Young Kim

Researcher Address: Severance Hospital, Yonsei-Ro 50-1, Seodaemun-Gu, Seoul, Korea

Phone: 02-2228-1992

Research nurse: Yoori Lim

Research nurse Address: Severance Hospital, Yonsei-Ro 50-1, Seodaemun-Gu, Seoul, Korea

Phone: 02-2228-4364 / 24 hours contact: 010-5353-4581

If you have any questions about your rights as a study participant, you can speak directly to the researcher or contact us at:

Severance Hospital, Institutional Review Board: Phone: 02-2228-0430-4

Severance Hospital, Hunan Research Protection Center: Phone: 02-2228-0450-4

**Informed Consent Form (Translated)**

| **Study Title:** | | Usefulness of non-contrast magnetic resonance imaging versus non-contrast ultrasonography for surveillance of hepatocellular carcinoma: prospective randomized study | | | | |
| --- | --- | --- | --- | --- | --- | --- |
| □ | I have read this document and have been explained about the study. I fully understand the purposes, procedures, benefits, and risks of the study, as well as alternative options available and policy for personal information. | | | | | |
| □ | I have asked all the questions and received the full answers to them. | | | | | |
| □ | I have been explained that I can withdraw my consent at any time even after agreeing to participate in this study, and that I can receive alternative standard treatments after the withdrawal. | | | | | |
| □ | I confirm that I have received one copy of the written consent form and explanation. | | | | | |
| □ | After due consideration, I agree to participate in this study on the basis of my free will. | | | | | |
|  | | | | | | |
| Participant’s name | | |  | Signature |  | Date (Y/M/D) |
| ■ Relationship with participant: | | | | | | |
| Surrogate’s name (if applicable) | | |  | Signature |  | Date (Y/M/D) |
|  | | | | | | |
| Observer’s name (if applicable) | | |  | Signature |  | Date (Y/M/D) |
|  | | | | | | |
| Investigator’s name | | |  | Signature |  | Date (Y/M/D) |
